# Revisiting spatial scale in the productivity–species richness relationship: fundamental issues and global change implications

**DOI:** 10.1093/aobpla/plu057

**Published:** 2014-09-23

**Authors:** Paul D. McBride, Jarrod Cusens, Len N. Gillman

**Affiliations:** Institute for Applied Ecology New Zealand, School of Applied Science, Auckland University of Technology, Private Bag 92006, Auckland, New Zealand

**Keywords:** Diversity, global change, macroecology, productivity, scale, species richness

## Abstract

The relationship between net primary productivity (NPP) and species richness has been the subject of long-running debate. A changing climate gives added impetus to resolving this debate, as it becomes increasingly necessary to predict biodiversity responses that might arise from shifts in productivity or its climatic correlates. It has become increasingly clear that at small scales productivity–species richness relationships (PSRs) are variable, while at macro scales relationships are typically positive. We demonstrate the importance of explicitly considering scale in discussions on PSRs even at large scales by showing that distinct patterns emerge in a global dataset of terrestrial ecoregions when ecoregions are binned into size classes. At all sizes, PSRs in ecoregions are positive, but the strength of the PSR scales positively with ecoregion size. In small ecoregions (10^3^–10^4^ km^2^), factors correlating with productivity play only a minor role in species richness patterns, while in large ecoregions (>10^5^ km^2^), NPP modelled from remotely sensed data is able to explain most of the variation in species richness. Better understanding the effects of scale on PSRs contributes to the debate on the relationship between species richness and productivity, which in turn allows us to better predict how both long- and short-term biodiversity patterns and ecosystem functioning might be altered under global change scenarios. This gives focus on future research to clarify causal pathways between species richness and productivity with appropriate attention to scale as an important focusing element.

## Abstract (precis)

The relationship between climate and biodiversity has been long debated. In a changing environment, there is new emphasis to resolve this debate for practical reasons: to manage conservation efforts we need to understand how diversity will change from both our own actions and natural global cycles. We show that the roles played by different ecological and evolutionary factors in shaping plant diversity change across the world's ecoregions, and—critically—that these differences scale with ecoregion size. Ecoregions that are both large and productive are globally important biodiversity sources that shape the biota of the smaller regions around them.

## Introduction

As a result of anthropogenic global change, species extinction is expected to become increasingly common in the next century (Şekercioğlu *et al.* 2004), with biodiversity losses themselves having local ecosystem impacts comparable with climate warming and nutrient pollution (Hooper *et al.* 2012). Biodiversity losses have a suite of drivers including habitat destruction, changing climate, nutrient deposition and exotic species introduction (Barnosky *et al.* 2011). While the anticipated effects of many global change facets are substantial, the full impacts are poorly understood as a number of fundamental questions remain incompletely answered. For example, there is considerable uncertainty around how plant net primary productivity (NPP) will respond to elevated atmospheric CO_2_ and associated warmer temperatures and shifting rainfall patterns (e.g. Norby and Zak 2011; Bader *et al.* 2013; De Kauwe *et al.* 2014). The extent to which plant species loss will affect NPP in terrestrial ecosystems is also unclear because the relationship between NPP and producer species richness can be both spatially (Whittaker *et al.* 2001; Chalcraft *et al.* 2004; Whittaker 2010) and temporally (Dodson *et al.* 2000; Cardinale *et al.* 2004) scale-dependent, as well as site- and taxon-specific (Dodson *et al.* 2000; Gough *et al.* 2000).

Compounding these complexities, the causal pathways that connect productivity and species richness are poorly understood (Adler *et al.* 2011; Grace *et al.* 2014). Not only might species losses impact productivity as has been observed at small scales, but global changes to climate that alter productivity through shifts in temperature, rainfall patterns and weather extremes may also have downstream effects on both plant and animal species richness. Indeed, species richness and productivity have both been experimentally manipulated to determine the influence of each on their bivariate relationship. It is now widely recognized that, at fine scales, as plant species richness increases so does productivity (Loreau *et al.* 2001). However, much of the evidence for this positive productivity–species richness relationship (PSR) comes from small-scale, fine-grain grassland experiments run over short time scales (e.g. Balvanera *et al.* 2006; Cardinale *et al.* 2011). At the same time, experimental manipulations of productivity (i.e. fertilization experiments) have often led to the seemingly contradictory position that small-scale PSRs are unimodal (Gough *et al.* 2000). However, fertilization can affect species richness as well as biomass production due to different stoichiometric requirements of competing species (Cardinale *et al.* 2009). Observational studies at similarly small spatial scales and in the same community types have found that PSRs are weak and inconsistent in pattern (Gillman and Wright 2006; Adler *et al.* 2011), presumably owing to a multitude of factors affecting small-scale species richness.

At larger regional and continental extents, at coarse grain sizes, among broad taxonomic groupings, and among both plants and animals, positive PSRs appear to predominate (Gillman and Wright 2006; Cusens *et al.* 2012), although the methods and results of synthetic reviews and meta-analyses on the topic have sparked widespread debate (see Gillman and Wright 2010; Gurevitch and Mengersen 2010; Lajeunesse 2010; Whittaker 2010 and related papers). In particular, while it is becoming increasingly clear that large-scale terrestrial PSRs are positive, it is unclear to what extent large- and small-scale patterns are congruent. Further, even when large-scale and small-scale studies recover similar forms of the PSR, they are unlikely to have the same causes. Firstly, as the grain size increases, the measure of diversity itself changes from alpha, or point, diversity to gamma diversity. Secondly, purely ecological mechanisms appear to be contributors rather than ultimate explanations of global diversity patterns (Rohde 1992; Currie *et al.* 2004). Instead, complex, emergent processes involving net diversification and historical contingency are likely to be increasingly important at larger scales, reflecting the influence of longer time scales on patterns at larger spatial scales (Whittaker *et al.* 2001). Despite this, small-scale ecological mechanisms have been invoked previously to explain global diversity patterns at the broadest scales. For example, Huston (1999 and references therein) argues that global plant diversity gradients can be explained by the local-scale mechanisms of competition and competitive exclusion.

Alternatively, productivity has been invoked to explain global gradients in species richness, and is among a suite of metrics related to water, energy and climate that are strongly correlated to macro-scale diversity. There are several alternate theories that invoke environmental energy or biologically available energy at large scales as a causal factor in shaping species richness patterns (e.g. the latitudinal diversity gradient). However, many of these theories are either unsupported by the available evidence or have yet to be clearly and mechanistically tested (Currie *et al.* 2004; Evans *et al.* 2005). As with smaller scales, it is unclear what is the link between species richness and key climatic factors such as temperature, rainfall, evapotranspiration and productivity. Because these variables correlate with each other, untangling cause from spurious correlation is challenging. Further, how these patterns change with scale remains incompletely explored.

We present a case study to draw attention to the central importance of scale when investigating PSRs even when working at very large scales. We take a dataset of global terrestrial ecoregions for which plant species richness estimates have been made, and calculate remotely sensed estimates of NPP for each to explore how PSRs vary with ecoregion size. We demonstrate that for ecoregions ranging from 1000–1 000 000 km^2^, the relationship between species richness and NPP varies in strength, scaling positively with ecoregion size. This changing relationship indicates that even at focal scales upwards of 1000 km^2^, the influence of factors regulating species richness is variable, and a consistently strong PSR only emerges across ecoregions >100 000 km^2^. We do not attempt to infer a direct causal relationship between species richness and productivity, here only examining the extent to which climate-related variables such as productivity correlate with species richness, and how this relationship changes across the scales investigated. Even between large (e.g. 10^3^ km^2^) and very large grains (e.g. 10^5^ km^2^), explicit consideration of scale is needed to interpret ecological relationships, and failure to do so affects our ability to resolve fundamental questions such as the causes of variable relationships between species richness and productivity. In turn, our ability to predict how biodiversity patterns might change as shifts in climate and other aspects of global change occur, and how ecosystems will respond to these changes, requires a consideration of scale.

Our results contribute to the fundamental understanding of global diversity patterns and their origins, as well as giving direction to future global change research. The spatial distribution of ecoregions, and their productivities, sizes and relations to other ecoregions inform us about their propensity for diversity. This information can become integrated in models predicting changes in biodiversity by including probable future shifts in primary productivity, as well as corresponding changes in ecoregion size and connectivity.

## Methods

### Data

We used terrestrial ecoregions (Olson *et al.* 2001) as our sampling units as they allow analysis of PSRs across a range of spatial scales spanning more than three orders of magnitude. Terrestrial ecoregions are biogeographic delineations of the earth's land surface intended for species conservation in the face of global change (Olson *et al.* 2001). The 867 described ecoregions separate distinctive biotas and regions of endemism by their natural boundaries. However, as Olsen *et al.* acknowledge, not all ecoregions are equally distinct, and expert's subjective opinion was the basis of their division. All systems for dividing ecological regions are subjective as they require weighting of competing factors, and the use of arbitrary limits and cut-off values, causing a degree of controversy over their adequacy (Omernik 2004), notably regarding their application to Indonesian conservation (Jepson and Whittaker 2002). Despite any limitations, the ecoregion concept is appealing and can provide at least a first-order approximation of its intended outcome. Kier *et al.* (2005) compiled estimates of floristic species richness for each of the 867 recognized terrestrial ecoregions (Olson *et al.* 2001). We accessed this dataset as well as GIS shapefiles for the ecoregions from http://worldwildlife.org/publications/terrestrial-ecoregions-of-the-world (WWF 2012) that we then used to estimate NPP for each ecoregion.

Net primary productivity data are available as either sparsely scattered point estimates that have been measured using a range of techniques (Clark *et al.* 2001), or as modelled estimates that are available at resolutions as fine as 0.1°. There has been a controversy surrounding the use of modelled NPP that has ranged from issues regarding inter-annual consistency (e.g. NDVI, see Diallo *et al.* 1991), through to assertions that, at a global scale, modelled NPP is fundamentally unrelated to true NPP because soil fertility is not considered and tropical NPP may be overestimated (Huston and Wolverton 2009). Some of the deficiencies in modelled NPP have been addressed by more recent algorithms that account for a broader range of environmental factors (see Zhao *et al.* 2005 and references therein). Indeed, without accounting for soil variation, current algorithms such as NASA's MODerate resolution Imaging Spectroradiometer (MODIS) products perform well when compared with direct estimates of NPP (Zhao *et al.* 2005), and while such estimates contain error they do not overestimate tropical NPP (Turner *et al.* 2006; Gillman *et al.* 2014). Further, although typically more accurate, direct measures of productivity are too sparse to provide predictions of NPP in ecoregions. Tropical estimates in particular of NPP have only recently been improving in scope and quality (e.g. Malhi *et al.* 2009). Therefore, because of the general congruence between measured and modelled NPP and the lack of spatial coverage for more direct NPP measurements, we have used modelled NPP in the present study. We downloaded NASA's MODIS MOD17A2 estimates of NPP for 2013 from http://neo.sci.gsfc.nasa.gov/view.php?datasetId=MOD17A2_M_PSN (NASA Earth Observations 2014). We then combined our data in a GIS using ArcGIS 10.1 and extracted mean values of NPP for each ecoregion as zonal statistics.

### Analysis

Overall patterns between species richness and both metrics were examined using ordinary least squares regression in R 3.0.2 (R Core Team 2014) for all ecoregions where estimates of NPP could be derived and species richness was above zero (*N* = 809). Although the terrestrial ecoregions vary from 6 to 4.6 × 10^6^ km^2^, most data fall between 1 × 10^3^ and 1 × 10^6^ km^2^ (758 ecoregions, 93.7 %). Within this data range, we binned ecoregions into orders of magnitude variation (e.g. 10^3^–10^4^ km^2^) to produce relatively homogeneous size classes within which species–area relationships (SARs) are substantially reduced. In addition, we added an extra class including only the largest ecoregions: those with area >10^5.5^ km^2^ (*N* = 115), which also includes the 16 ecoregions >10^6^ km^2^. While across the dataset, 17.6 % of variation in species richness is explained by area on a log–log scale (8.8 % on a semi-log scale with untransformed species richness), area did not explain >2 % of variation in species richness in any of our defined size classes.

**Figure 1.**
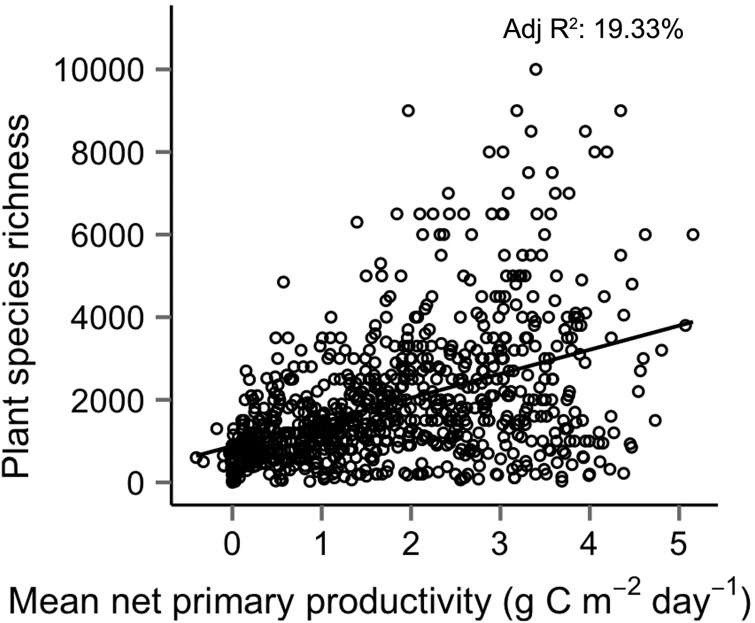
Relationship between modelled NPP and plant species richness of global terrestrial ecoregions (*N* = 781). Plant species richness estimates for terrestrial ecoregions from Kier *et al.* (2005). Net primary productivity is 2013 mean estimates for MOD17 modelled NPP (NASA 2014).

**Figure 2.**
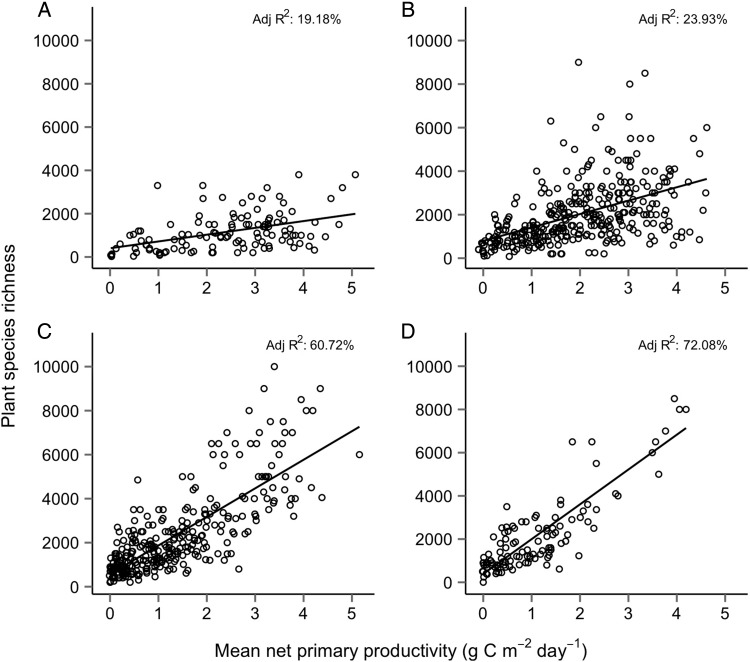
Relationship between modelled NPP and plant species richness across size classes of global terrestrial ecoregions: (A) small ecoregions between 10^3^ and 10^4^ km^2^ (*N* = 117); (B) medium ecoregions between 10^4^ and 10^5^ km^2^ (*N* = 339); (C) large ecoregions between 10^5^ and 10^6^ km^2^ (*N* = 302); and (D) the largest ecoregion subset (>10^5.5^ km^2^) (*N* = 115). Data sources as in the Fig. 1 caption.

We performed several checks to ensure that comparisons across size classes were not confounded by other variables. First, Kier *et al.* (2005) provide data quality measures for their estimates, depending on what type of information was used to derive them. In the case of the ‘poor’ and ‘very poor’ estimates, species richness values are indirect and some have been partially derived with climate information. The distribution of the four quality categories was unequal across our size classes, with higher average quality in the largest size class (10^5^–10^6^ km^2^), and lower average quality in the middle size class. Therefore, we compared analyses on the top and bottom two quality categories, and found that results were close to identical **[see Supporting Information]**. Second, the analysis would be weakened if a full range of productivity was not represented in each of the size classes. However, the range of productivity in all bins was similar (Figs 1 and 2). Third, we included realm and biome information in analyses to account for the possible confounding influence from these sources, whether through historical effects or from differences caused by sampling biases (i.e. more direct and accurate estimates of species richness in well-studied northern temperate ecoregions).

Lastly, we further subdivided our ecoregion size classes into 12 bins based on log-transformed area to better determine the extent to which the strength of the PSR scales with ecoregion size. Bins contained between 9 and 110 ecoregions (median bin size: 61 ecoregions). For each bin, the PSR was determined, and then the relationships were examined across these bins. We compared the relationship for productivity with that for latitudinal midpoints of ecoregions.

## Results

Taken as a whole, there is a weak, positive relationship between productivity and species richness across terrestrial ecoregions (Fig. 1; adjusted *R*^2^ = 19.4 %). While mean species richness is greater in regions where NPP is higher, species richness in highly productive ecoregions varies from hundreds of species to 10 000 species. Hence, while productivity correlates with maximum species richness, unrelated factors determine actual richness at or below that level.

However, the strength of PSRs varies systematically with ecoregion size. Amongst small ecoregions (10^3^–10^4^ km^2^, *N* = 117), NPP is a poor predictor of species richness (Fig. 2A; adjusted *R*^2^ = 19.2 %). In addition, maximum species richness in these smaller ecoregions is 3800 species, indicative of a possible limit imposed by available area. Medium ecoregions (10^4^–10^5^ km^2^, *N* = 339) display a PSR in which NPP has a modestly improved predictive ability (Fig. 2B; adjusted *R*^2^ = 23.9 %). Peak species richness is also substantially greater, at 90 % of the global maximum ecoregion species richness. However, similar to the combined analysis of all ecoregions, at peak productivity the lower limit of species richness was indistinguishable from that found in low productivity ecoregions, indicative of unmeasured constraints that are not related to NPP. Amongst large ecoregions (10^5^–10^6^ km^2^, *N* = 302), these unmeasured constraints on species richness were no longer evident, and the PSR was strong (Fig. 2C; adjusted *R*^2^ = 60.7 %). Finally we repeated the analysis with only the largest ecoregions in the dataset (log_10_ area >5.5, *N* = 115) and found a strong, monotonic positive relationship (Fig. 2D; adjusted *R*^2^ = 72.3 %).

**Figure 3.**
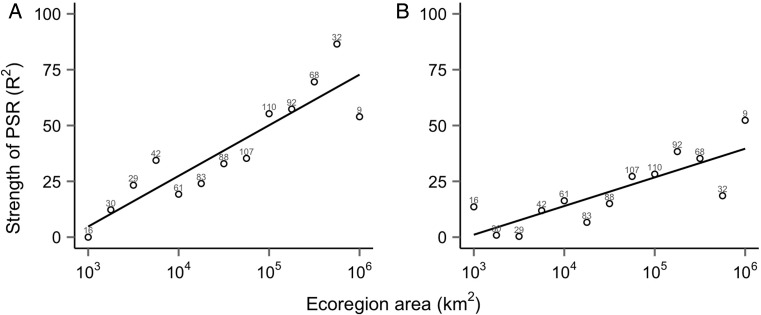
Strength of relationship (linear regression-adjusted *R*^2^) between species richness and (A) modelled mean annual NPP; and (B) ecoregion latitudinal midpoints, across ecoregions ranging from 10^3^ to 10^6^ km^2^, binned by size into 0.25 increments on a logarithmic scale (i.e. Bin 1: 10^3^–10^3.25^ km^2^). Numbers above points indicate the ecoregion count within that size class. Data sources as in the Fig. 1 caption.

When binned into narrower size classes, the strength of PSRs scales closely with ecoregion size. In the smallest ecoregions, productivity and species richness do not appear to co-vary (adjusted *R*^2^ = 0 %), but the strength of the relationship increases monotonically as the ecoregion size increases, peaking in the second largest size class bin (adjusted *R*^2^ = 86.4 %) (Fig. 3A). When latitude replaced productivity as a predictor of species richness, the pattern was qualitatively similar, but the strength of relationship was weaker.

Because some lower-quality estimates of species richness made by Kier *et al.* (2005) were partially derived from SARs, we tested for an effect of quality on the derived patterns. Relationships were almost identical both qualitatively and quantitatively when the data were restricted to either only the upper quality categories or the lower quality categories, indicating that the results are not dependent on estimates of species richness that may have partially derived from climate variables or SARs [**see Supporting Information**].

Differences in species richness also exist between realms and biome types. Indeed, biome type and realm predict ecoregion species richness within ecoregion size classes, and explanatory power increases in the larger ecoregion size classes (Table 1). In small and medium ecoregions, biome type and realm explain approximately twice the variation in species richness explained by NPP. In small ecoregions, area did not contribute to preferred models, and was non-significant in every model. Evidence ratios from Akaike weights (Burnham and Anderson 2002; Wagenmakers and Farrell 2004) derived from the bias-corrected Akaike Information Criterion (AICc) indicated that NPP + realm + biome was the best fit model in the smallest ecoregions (Table 1). In medium ecoregions, area slightly but significantly improved the relationship, and the preferred model was the most complex: NPP + realm + biome + area. Indeed, even in the smaller and medium-sized ecoregion size classes, where NPP is a poor predictor of species richness, this model explained approximately half of the variation in species richness. In large ecoregions, the inclusion of realm was not favoured by AICc. However, area was retained in the preferred model (Table 1).

**Table 1.** Akaike Information Criterion-based model selection among linear regression models of the productivity–species richness relationships within terrestrial ecoregion size classes, controlling for log_10_area, realm and biome type. Evidence ratios are provided for the ratio of Akaike weights between the first and second models. Data sources as in the Fig. 1 caption. All tested models are included. Variables marked with an asterisk were not significant in that model.

	Adjusted *R*^2^ (%)	Δ_AICc	AICc weight	Evidence ratio
**Small ecoregions, 10^3^–10^4^ km^2^**				
NPP + realm + biome	48.91	0	0.53	3.61
Realm + biome	47.32	1.73	0.22	
NPP + realm + biome + area*	48.64	2.57	0.15	
Realm + biome + area*	47.42	3.38	0.01	
NPP + biome	38.82	8.99	<0.01	
NPP + biome + area*	39.64	9.01	<0.01	
NPP + realm	27.29	20.50	<0.01	
NPP + realm + area*	26.81	22.63	<0.01	
NPP	19.18	24.37	<0.01	
NPP + area*	19.20	25.46	<0.01	
**Medium ecoregions, 10^4^–10^5^ km^2^**				
NPP + realm + biome + area	53.26	0	>0.99	1.4 × 10^9^
Realm + biome + area	50.75	16.44	<0.01	
NPP + realm + biome	47.60	37.49	<0.01	
NPP + biome + area	45.60	44.15	<0.01	
Realm + biome	45.78	47.86	<0.01	
NPP + realm + area	39.15	74.17	<0.01	
NPP + biome	38.57	84.22	<0.01	
NPP + area	32.51	102.87	<0.01	
NPP + realm	31.51	113.16	<0.01	
NPP	23.93	142.38	<0.01	
**Large ecoregions, 10^5^–10^6^ km^2^**				
NPP + biome + area	70.37	0	0.45	1.06
NPP + realm + biome + area	70.95	0.12	0.42	
NPP + realm + biome	70.49	3.62	0.07	
NPP + biome	69.85	4.10	0.06	
Realm + biome	64.53	57.96	<0.01	
Realm + biome + area	64.56	58.95	<0.01	
NPP + realm + area	62.42	63.81	<0.01	
NPP + realm	62.03	65.77	<0.01	
NPP + area	61.15	68.41	<0.01	
NPP	60.72	70.68	<0.01	
**Largest ecoregions, >10^5.5^ km^2^**				
NPP + biome + area	79.04	0	0.93	15.92
NPP + biome	77.73	5.54	0.06	
NPP + realm* + biome + area	79.23	8.72	0.01	
NPP + realm + biome	77.75	14.92	<0.01	
NPP + area	73.04	15.91	<0.01	
NPP	72.08	18.78	<0.01	
NPP + realm* + area	73.65	20.68	<0.01	
NPP + realm*	72.70	23.45	<0.01	
Realm + biome	67.77	55.84	<0.01	
Realm + biome + area	68.14	56.22	<0.01	

Across all ecoregion size classes, the main biome type was tropical and subtropical moist broadleaf forests. However, biome proportions varied across size classes. To remove possible variation in the strength of relationships across size classes resulting only from changes in biome proportions, we analysed the PSRs in tropical and subtropical moist broadleaf forest ecoregions separately (*N* = 225). The overall predictive ability of the model was reduced, especially among small and medium ecoregions (*R*^2^ < 7 %), while the qualitative pattern of increasing model strength with ecoregion size was upheld. The PSR in the largest ecoregions remained strong (Table 2, adjusted *R*^2^ = 57.6 %).

**Table 2.** Comparison of linear regression models of plant species richness and NPP for all terrestrial ecoregions (*N* = 809), and the subset of tropical and subtropical moist broadleaf forest ecoregions (*N* = 225) across four ecoregion size classes. Data sources as in the Fig. 1 caption.

Ecoregion size (km^2^)	Adjusted *R*^2^ (%)	
	All biomes	Moist broadleaf forests
10^3^–10^4^	19.2	0
10^4^–10^5^	23.9	6.9
10^5^–10^6^	60.7	20.6
>10^5.5^	72.3	57.6

## Discussion

The prospect of understanding biodiversity within a global change context presents the challenge of not simply studying static diversity patterns as they exist now, but investigating the causes of those patterns so that responses to natural and anthropogenic changes can be predicted. A scale-dependent understanding of PSRs is an important step towards achieving this goal, as the additional information allows for more detailed and context-specific predictions to be made.

Here, we have focused on exploring PSRs using terrestrial ecoregions. Terrestrial ecoregions provide a set of floristically distinguishable vegetation patches across the world that range in size by several orders of magnitude. Because their species compositions differ from their neighbours, when treated as sampling units they have greater independence than would large-grain samples across landscapes falling within the same ecoregion. As such, ecoregions can provide insights into processes that occur at different spatial scales. We show that the strength of PSRs is dependent on ecoregion size, demonstrating the importance of scaling factors in the determinants of species richness patterns, and the centrality of scale in the long-running debate on the nature of macro-scale PSRs.

While the overall PSR across all terrestrial ecoregions is poor, which could imply that a generally weak relationship exists between species richness and productivity, analysis of ecoregions binned into size classes reveal a distinct scaling effect of area. In small and medium ecoregions (10^3^–10^5^ km^2^), NPP has a weak relationship with plant species richness, while in large ecoregions (>10^5^ km^2^), NPP predicts a substantial amount of the variation in plant species richness. When binned into narrower size classes, the strength of the PSR scales linearly with ecoregion size (Fig. 3A), indicating a central role for area in determining the importance of factors that affect species richness. Because there is an interaction between ecoregion size and the strength of the PSR, including ecoregion size as a covariate is not sufficient to account for the influence of ecoregion size on PSRs. Doing so improves the model fit across the whole dataset (linear regression: species richness ∼ NPP + log_10_ area, adjusted *R*^2^ = 43.0 %) but fails to capture the heterogeneity in the relationship where productivity accounts for almost no variation in species richness in small ecoregions to three-quarters in the largest ecoregions (Fig. 3A).

Reconciling the varying nature of PSRs across scales requires consideration of the ecological and evolutionary dynamics that occur at these different scales. Small ecoregions—from 10^3^ to 10^4^ km^2^—are equivalent to circular areas with radiuses from 17.8 to 56.4 km. As such, it is unlikely that many existing species in regions of this size would be the result of an accumulation of lineages diversifying within the ecoregion, although areas sufficiently isolated by distance or environment (e.g. islands and montane regions) are likely to contain endemics that have diverged from species in the regional pool. The species richness dynamics of small ecoregions are likely to be controlled by the constraints of their size and ecological factors such as regional species pools, aspects of site history such as disturbance, and dispersal rates from neighbouring regions with compatible floras. In other words, their PSRs are likely to be weak for the same reasons that PSRs have been found to be weak in other small scale studies—the confluence of myriad ecological factors, many of which are unrelated to productivity. Area was non-significant in models predicting small ecoregion species richness (Table 1). The preferred model included NPP, biogeographic realm and biome type as predictors, although this model was only moderately preferred over the latter two predictors excluding NPP. The apparent upper limit on species richness in these ecoregions (Fig. 2A) could be a result of their size—given similar alpha diversity and turnover, smaller ecoregions will have fewer species.

Compared to smaller ecoregions, the species composition of large ecoregions (>10^5^ km^2^) is likely to more strongly reflect diversification processes occurring within the ecoregion, as indicated by the increasingly strong relationship with NPP. There is a long-recognised correlation between environmental/biologically available energy, of which productivity is a measure, and global-scale species richness in both plants and animals (Hawkins *et al.* 2003; Evans *et al.* 2005; Gillman and Wright 2006; Cusens *et al.* 2012). Unlike small and medium ecoregions where realm is an important predictor of species richness, only biome type, NPP and ecoregion area were included in the preferred model predicting large ecoregion species richness. The unimportance of biogeographic realm in predicting species richness is suggestive of a reduced role for history and contingency in large ecoregions. Further, in the largest ecoregions (>10^5.5^ km^2^), NPP alone predicts more than 70 % of variation in species richness (Table 1), suggesting a close relationship with net diversification. We make no inference here about whether productivity or the climatic correlates of productivity are most closely associated with changes in net diversification, as causal pathways are yet to be determined. However, we note that it has recently been shown that climate variables (e.g. temperature and rainfall) outperform productivity as predictors of species richness in forests at the global scale (Šímová *et al.* 2011).

Medium ecoregions differ from small ecoregions by having a less clear upper limit on diversity: species richness in these ecoregions peaks at levels similar to that of large ecoregions. Unlike small ecoregions, the inclusion of NPP in the model predicting species richness was substantially more strongly favoured in medium ecoregions (evidence ratio 1.4 × 10^9^, Table 1). Also different from the other ecoregion size classes, the most complex model predicting species richness was strongly favoured in medium ecoregions (Table 1). NPP's predictive ability for species richness was low (bivariate *R*^2^ = 23 %, Table 1); at moderate and high productivities substantial variation is observable in the species richness of ecoregions (Fig. 2B). We discuss the biogeographic distribution of these ecoregions below.

To further investigate PSR scaling effects, we performed a post-hoc analysis examining how ecoregion PSRs within size classes varied with latitude **[see Supporting Information]**. Ecoregions of all sizes have tropical diversity peaks and approximately symmetrical northern and southern hemisphere declines in species richness as the latitude increases. However, only large ecoregions have distinctly negative relationships between NPP and absolute latitude; small and medium ecoregions have strong hemispheric biases, with the latter in particular showing little co-variation between latitude and NPP in the southern hemisphere **[see Supporting Information]**. Indeed, with the exception of one North American ecoregion, medium size class ecoregions that were both high productivity (>4 g C m^−2^ day^−1^) and relatively low species richness (<2000 species) were located in the Australasian realm, either as oceanic islands or parts of New Zealand. This distribution indicates a biogeographic basis for the high level of variance in species richness at high productivity sites. Isolated parts of Australasia may harbour smaller regional species pools to colonize smaller ecoregions, placing different constraints on species richness levels than those experienced elsewhere.

Only in the large ecoregion size class does NPP follow a symmetrical latitudinal gradient, while species richness follows approximately symmetrical latitudinal gradients across all size classes **[see Supporting Information]**. One interpretation of these observations is that species richness patterns are derived from factors that co-vary with latitude but are unconnected to NPP or other energy correlates directly. If true, the scaling PSR could be an artefact of a general latitudinal gradient in NPP. However, NPP is a substantially better predictor of species richness than is latitude, particularly for ecoregions >10^5^ km^2^ (Fig. 3). Factors relating to NPP therefore appear to play a primary role in determining species richness, while the latitudinal diversity gradient across smaller ecoregions is consistent with their floras being derived primarily from larger, neighbouring ecoregions.

We are far from the first to emphasize the role of scale in the study of diversity patterns, in particular relating to PSRs (e.g. Whittaker *et al.* 2001; Chase and Leibold 2002; Chalcraft *et al.* 2004; Gillman and Wright 2006; Whittaker 2010). Nonetheless, there remains a tendency to conflate small- and large-scale mechanisms that shape diversity patterns. For example, Huston (1999) argues that diversity patterns ranging from scales of a few metres to global diversity gradients can be explained by the intensity of competition and competitive exclusion, a mechanism that is theorized to operate on alpha diversity among interacting individuals (Grime 1973; Huston 1979), and predicts a unimodal PSR. More recently, Huston and Wolverton (2009) have suggested that NPP may be lower in tropical than temperate zones, placing the latitudinal species richness gradient in a framework implying a global unimodal PSR. Their model places primacy on soil fertility and points out that many measures of NPP are indirect. However, directly measured annual neotropical productivity is substantially higher than the annual temperate NPP (e.g. Aragão *et al.* 2009) and is in generally good agreement with modelled NPP derived from remotely sensed data (Zhao *et al.* 2005). Correspondingly, a recent study of latitudinal patterns of directly measured NPP found a monotonic decline in NPP with increasing latitude (Gillman *et al.* 2014). In any case, such a universal mechanism proposed by Huston (1999) is unlikely to exist, as our results suggest that the nature and strength of PSRs change with grain size and extent (see also Whittaker *et al.* 2001; Whittaker and Heegaard 2003; Gillman and Wright 2006). Indeed, the importance that climate and productivity have in predicting species richness decreases at smaller grains and extents whereas factors such as habitat heterogeneity and edaphics/nutrients become more important at smaller grains and extents (Field *et al.* 2009).

We note that the recent literature on the PSR has emphasized the importance of exploring relationships based on causal mechanisms through techniques such as structural equation modelling, rather than identifying bivariate correlations (Adler *et al.* 2011; Grace *et al.* 2014). We agree that such approaches are critical to moving forward in our understanding of PSRs and we add to this the importance of scale whenever investigating these relationships. Work on fine-scale patterns in grasslands has greatly improved our knowledge of PSRs. However, with such a strong reliance on a single biome there is the risk of overgeneralizing results. For example, while we agree with Adler *et al.* (2011) that productivity is a poor predictor of species richness in small-grain, temperate grassland plots, we would caution against generalizing from this that productivity is a poor predictor of species richness wholesale. Instead, we reiterate Adler *et al.*'s timely suggestion to look more deeply for causal mechanisms in species richness patterns.

Finally, PSRs are ecologically important in the context of a changing climate. While small-grain studies reveal complex PSRs occurring on short time scales, at large grains species richness patterns appear to obey more predictable laws over longer time scales. The implications of species loss from global change can thus be considered on two levels: the direct effects of species loss on ecosystem functioning, occurring on short time scales and local spatial scales; and the long-term prospects for recovery of global diversity. On the first level, the short-term, local effects of losing species may be variable—perhaps on average resulting in lower productivity (Hooper *et al.* 2012) and loss of ecosystem services (Cardinale *et al.* 2012). On the second level, it appears that the prospects for recovery of global diversity may rely on the species pools harboured in large ecoregions. Isolated, small or low-productivity ecoregions not only contain fewer species, but their patterns of species richness appear more closely tied to the those of surrounding large ecoregions rather than the climate correlates that have previously been linked to diversification rates.

## Conclusions

Productivity and species richness have a complex relationship that varies in strength with spatial scale. While it has previously been shown that scale is important in PSRs at the local-to-landscape level, we demonstrate here that even with much larger sampling units, scaling effects are not only detectable but strong. Our results suggest caution when investigating species richness patterns across ecoregions if assuming a homogeneous relationship exists with causal factors—for example, if studying species richness along a productivity gradient. Moving forward in understanding species richness patterns will require clearer thinking about the multitude of causes of diversification patterns, and the scales on which they act and interact. Moving forward will also require more sophisticated casual modelling at large scales, in line with the recent direction at small scales. Long-term protection of global biodiversity requires a better understanding of the flow of species and heterogeneity in diversification across ecoregions, as well as predictive modelling of the global changes that will affect the climates and boundaries of ecoregions.

## Sources of Funding

P.M. is supported by a doctoral scholarship from the Institute for Applied Ecology New Zealand. J.C. is supported by an Auckland University of Technology Vice Chancellor's Doctoral Scholarship.

## Contributions by the Authors

P.D.M. and J.C. conceived the idea. P.D.M. analysed the data. All authors wrote the manuscript.

## Conflicts of Interest Statement

None declared.

## Supporting Information

The following Supporting Information is available in the online version of this article –

**Figure S1.** Relationship between modelled net primary productivity (NPP) and plant species richness across size classes of global terrestrial ecoregions for ecoregions with upper quality estimates (quality Categories 1 and 2 in Kier *et al.* 2005): (A) small ecoregions between 10^3^ and 10^4^ km^2^ (*N*= 51); (B) medium ecoregions between 10^4^ and 10^5^ km^2^ (*N*= 108); (C) large ecoregions between 10^5^ and 10^6^ km^2^ (*N*= 160); and (D) the largest ecoregion subset (>10^5.5^ km^2^) (*N*= 70). Net primary productivity is 2013 mean estimates for MOD17 modelled NPP (NASA 2014), full data URL in the main text.

**Figure S2.** Relationship between modelled NPP and plant species richness across size classes of global terrestrial ecoregions for ecoregions with lower quality estimates (quality Categories 3 and 4 in Kier *et al.* 2005): (A) small ecoregions between 10^3^ and 10^4^ km^2^ (*N*= 66); (B) medium ecoregions between 10^4^ and 10^5^ km^2^ (*N*= 231); (C) large ecoregions between 10^5^ and 10^6^ km^2^ (*N*= 142); and (D) the largest ecoregion subset (>10^5.5^ km^2^) (*N* = 45). Data sources in Figure S1.

**Figure S3.** Latitudinal patterns of species richness and NPP in: (A) small ecoregions; (B) medium ecoregions and (C) large ecoregions. NPP data are divided into quartiles for each bin. Data sources in Figure S1.

Additional Information
