# Supplementary material for: Revisiting spatial scale in the productivity–species richness relationship: fundamental issues and global change implications
Source: AoB Plants. 2014 Sep 23;6:plu057. doi: 10.1093/aobpla/plu057 (PMC4231355; doi:10.1093/aobpla/plu057)
Supplement: Additional Information [file supp_6_plu057_index.html]

Revisiting spatial scale in the productivity–species richness relationship: fundamental issues and global change implications — Additional Information 

# Revisiting spatial scale in the productivity–species richness relationship: fundamental issues and global change implications

## Additional Information

Additional Information

**Files in this Data Supplement:**

- Supporting Information - docx file
