# Supplementary material for: Revisiting spatial scale in the productivity–species richness relationship: fundamental issues and global change implications
Source: AoB Plants. 2014 Sep 23;6:plu057. doi: 10.1093/aobpla/plu057 (PMC4231355; doi:10.1093/aobpla/plu057)
Supplement: Additional Information [file supp_plu057_plu057supp.docx]

**‘Revisiting spatial scale in the productivity–species richness relationship’ AoB Plants 14078 Supporting Information**

**Effects of data quality**

To determine if species richness estimate quality affected our results, we divided our data into the top and bottom quality estimates. The upper quality estimates formed 44% of the dataset, while 56% were lower quality estimates. We repeated our main analysis on these subsets to determine if the strength of relationships across size classes was an artifact of data quality. Results fir the upper quality estimates (Figure S1) and lower quality estimates (Figure S2) are congruent, and comparable to those presented in Figure 2 in the main text for the entire dataset.


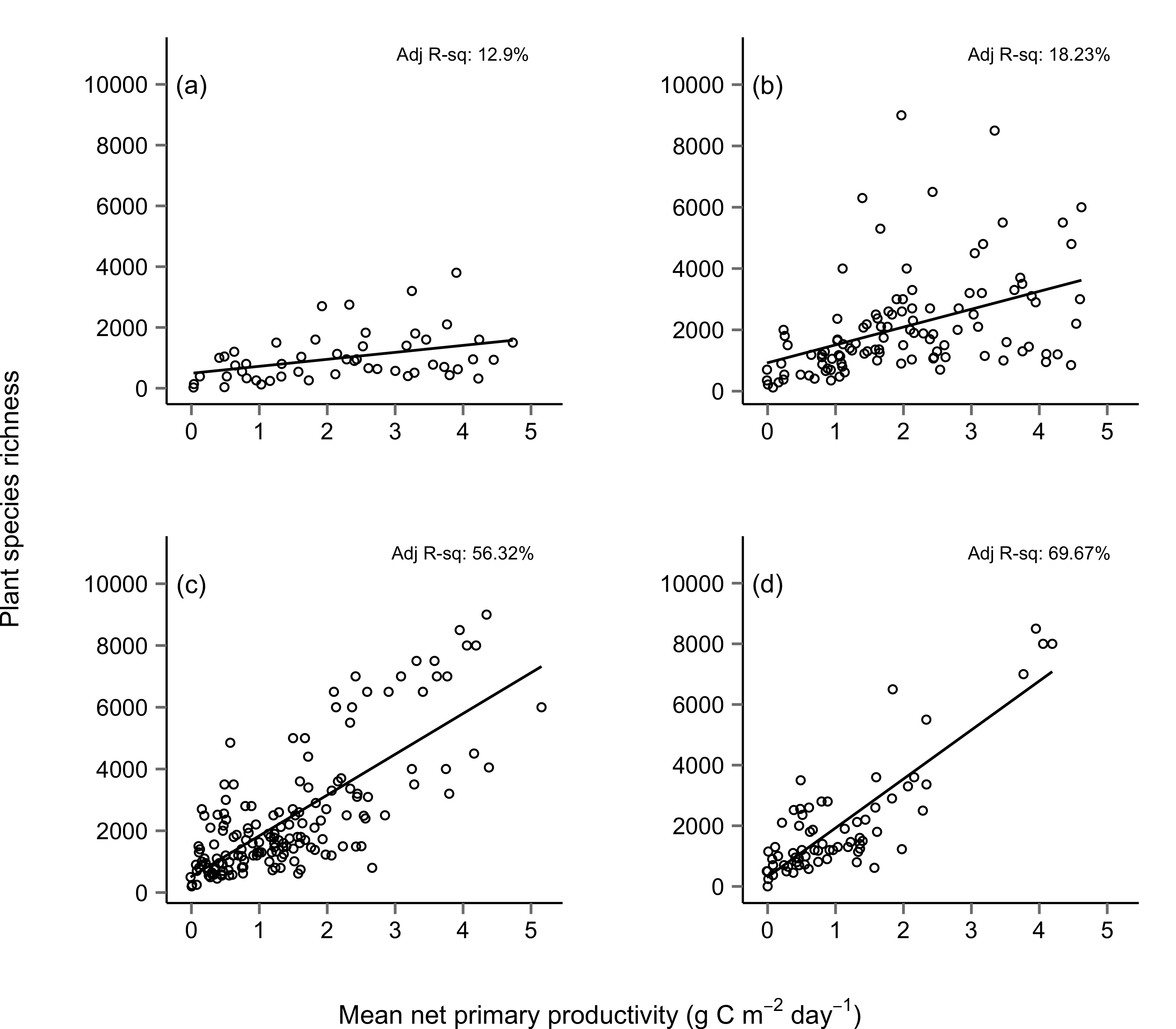


**Figure S1.** Relationship between modelled net primary productivity and plant species richness across sizes classes of global terrestrial ecoregions for ecoregions with upper quality estimates (quality categories 1 and 2 in Kier *et al*. 2005): (a) small ecoregions between 10^3^ and 10^4^ km^2^ (N = 51); (b) medium ecoregions between 10^4^ and 10^5^ km^2^ (N = 108); (c) large ecoregions between 10^5^ and 10^6^ km^2^ (N = 160); and (d) the largest ecoregion subset (>10^5.5^ km2) (N = 70). Net primary productivity is 2013 mean estimates for MOD17 modelled NPP (NASA 2014), full data URL in main text.


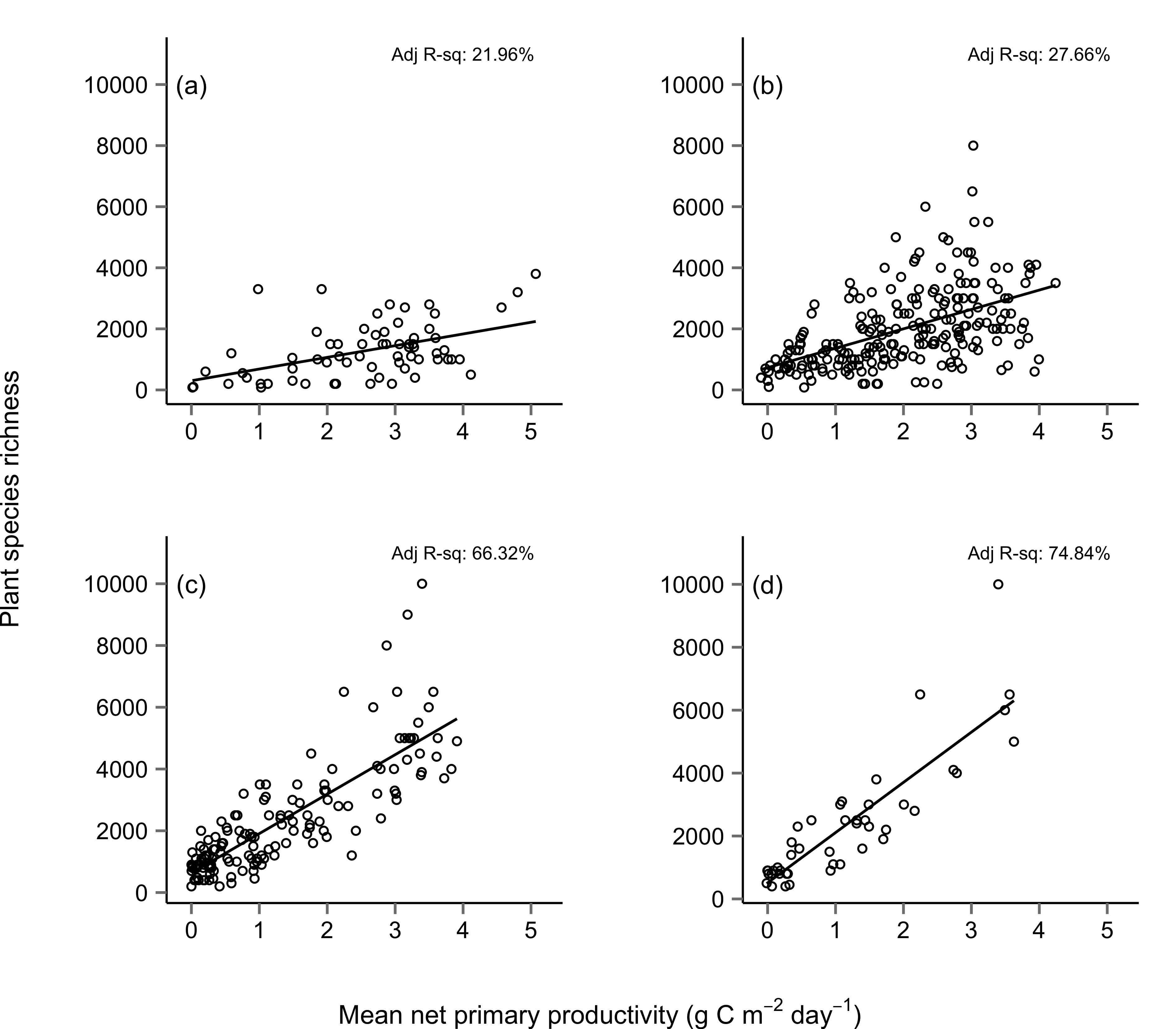


**Figure S2.** Relationship between modelled net primary productivity and plant species richness across sizes classes of global terrestrial ecoregions for ecoregions with lower quality estimates (quality categories 3 and 4 in Kier *et al*. 2005): (a) small ecoregions between 10^3^ and 10^4^ km^2^ (N = 66); (b) medium ecoregions between 10^4^ and 10^5^ km^2^ (N = 231); (c) large ecoregions between 10^5^ and 10^6^ km2 (N = 142); and (d) the largest ecoregion subset (>10^5.5^ km^2^) (N = 45). Data sources in Figure S1.

**Latitudinal trends in species richness and NPP**

To determine possible reasons for smaller and moderately sized ecoregions having poor or heteroscedastic relationships between species richness and net primary productivity, we investigated how these relationships co-varied with latitude within the three ecoregion size classes. The ecoregions within each size class followed a distinct latitudinal diversity gradient. However, while NPP peaks in the tropics, there is an asymmetry in its decline with increasing latitude (Figure S3). This asymmetry is most obvious in moderately sized ecoregions, which is also the data subset with the greatest heteroscedasticity (Figure 2b in main text). Northern ecoregions show on average both declining NPP and species richness with increasing latitude, while NPP is relatively insensitive to latitude in the southern hemisphere.

The larger ecoregions have both a more symmetric latitudinal gradient in NPP, and a more distinctly unimodal (rather than hump-backed) species richness gradient. The larger ecoregions also display an asymmetry in numbers of north and southern ecoregions. Such a pattern is expected because of the hemispheric land distribution.


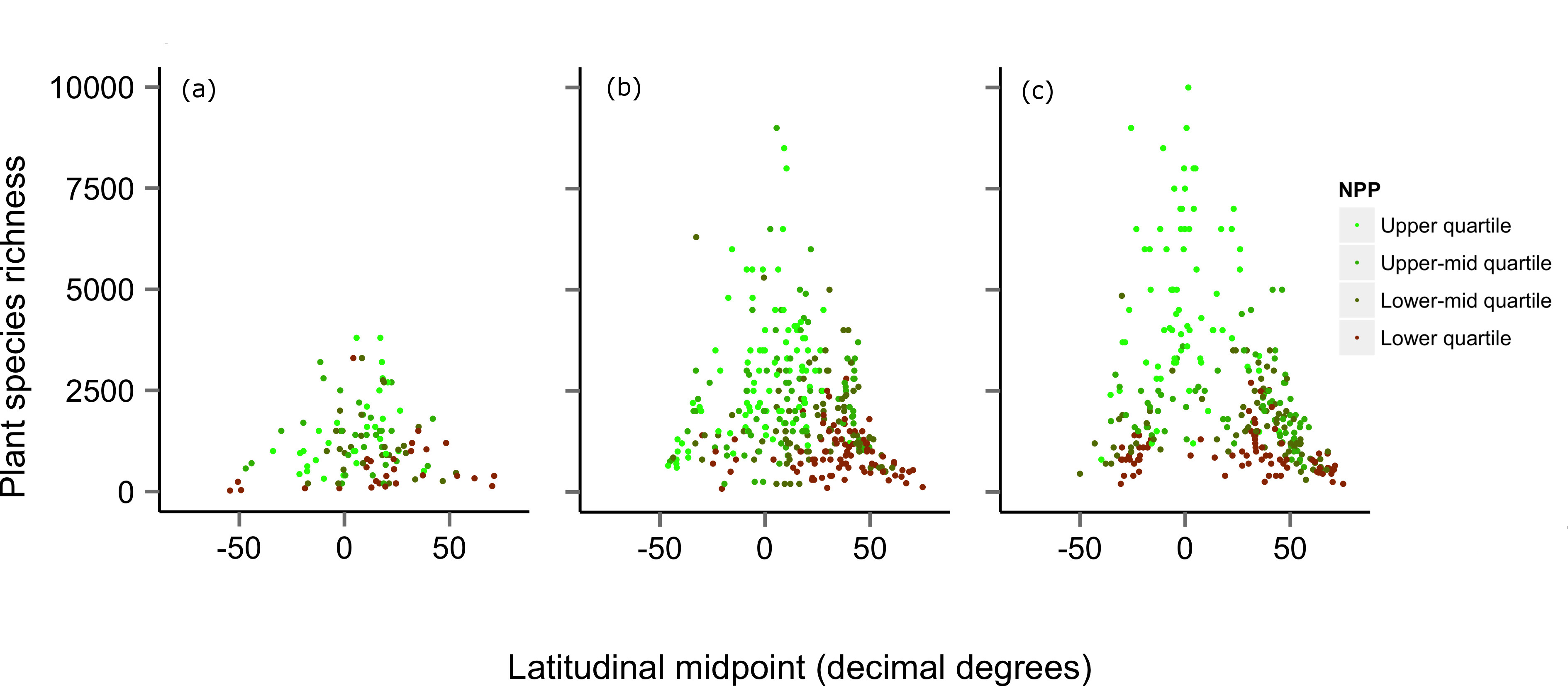


**Figure S3.** Latitudinal patterns of species richness and net primary productivity in: (a) small ecoregions (b) medium ecoregions and (c) large ecoregions. NPP data are divided into quartiles for each bin. Data sources in Figure S1.

**REFERENCES**

Kier G, Mutke J, Dinerstein E, Ricketts TH, Küper W, Kreft H, Barthlott W. 2005. Global patterns of plant diversity and floristic knowledge. Journal of Biogeography, 32: 1107-1116.

NASA Earth Observations. 2014. Net primary productivity (1 month - Terra/MODIS).
